# The EU chemicals strategy for sustainability: in support of the BfR position

**DOI:** 10.1007/s00204-021-03125-w

**Published:** 2021-08-07

**Authors:** Frank A. Barile, Sir Colin Berry, Bas Blaauboer, Alan Boobis, Herrmann M. Bolt, Christopher Borgert, Wolfgang Dekant, Daniel Dietrich, Jose L. Domingo, Corrado L. Galli, Gio Batta Gori, Helmut Greim, Jan G. Hengstler, Pat Heslop-Harrison, Sam Kacew, Hans Marquardt, Angela Mally, Olavi Pelkonen, Kai Savolainen, Emanuela Testai, Aristides Tsatsakis, Nico P. Vermeulen

**Affiliations:** 1 College of Pharmacy and Health Sciences, St John’s University, Queens, NY USA; 2 Queen Mary University of London, London, UK; 3 Division of Toxicology, Institute for Risk Assessment Sciences, Utrecht University, Utrecht, The Netherlands; 4 National Heart and Lung Institute, Imperial College, London, UK; 5 Leibniz Research Centre for Working Environment and Human Factors (IfADo), TU Dortmund, Dortmund, Germany; 6 Applied Pharmacology and Toxicology, Inc., Gainesville, FL USA; 7 Department of Toxicology, University of Würzburg, Würzburg, Germany; 8 Faculty of Biology, University of Konstanz, Konstanz, Germany; 9 Laboratory of Toxicology and Environmental Health, School of Medicine, IISPV, Universitat ‘Rovira I Virgili’, Reus, Spain; 10 Department of Pharmacological and Biomolecular Sciences, University of Milan, Milan, Italy; 11 The Health Policy Center, Bethesda, MD USA; 12 Technical University of Munich, Munich, Germany; 13 Department of Genetics and Genome Biology, University of Leicester, Leicester, UK; 14 McLaughlin Centre for Risk Assessment, University of Ottawa, Ottawa, Canada; 15 Toxicology, University of Hamburg, Hamburg, Germany; 16 Department of Pharmacology and Toxicology, University of Oulu, Oulu, Finland; 17 Nanosafety Research Centre, Finnish Institute of Occupational Health, Helsinki, Finland; 18 Environment and Health Department, Instituto Superiore Di Sanità, Rome, Italy; 19 Medical School, University of Crete, Crete, Greece; 20 Department of Chemistry and Pharmaceutical Sciences, Vrije Universiteit, Amsterdam, The Netherlands

## Abstract

The EU chemicals strategy for sustainability (CSS) asserts that both human health and the environment are presently threatened and that further regulation is necessary. In a recent Guest Editorial, members of the German competent authority for risk assessment, the BfR, raised concerns about the scientific justification for this strategy. The complexity and interdependence of the networks of regulation of chemical substances have ensured that public health and wellbeing in the EU have continuously improved. A continuous process of improvement in consumer protection is clearly desirable but any initiative directed towards this objective must be based on scientific knowledge. It must not confound risk with other factors in determining policy. This conclusion is fully supported in the present Commentary including the request to improve both, data collection and the time-consuming and bureaucratic procedures that delay the publication of regulations.

## Commentary

The declared objective of the EU Chemicals Strategy for Sustainability (CSS) is to “restore human health and environment to a good quality status” with respect to “substances of concern” (European Commission 2019). Examples, where it is thought by the CSS that there is concern and a need for better data, include exposures of vulnerable groups, endocrine-disrupting chemicals, and the risk assessment of mixtures.

In their Guest Editorial “EU chemicals strategy for sustainability questions regulatory toxicology as we know it: is it all rooted in sound scientific evidence?” published in this journal, several members of the German competent authority for risk assessment, the Federal Institute for Risk Assessment (BfR), raise concerns about the scientific justification for the CSS (Herzler et al 2021). We, as representatives of the international scientific community^1^ appreciate, that a competent authority, which stands for a science-based risk assessment of chemicals, may properly express concern about the need to “restore human health and environment to a good quality status”. We are concerned that scientific data appear to be used as a justification for an intervention whereas the basis is “public concern”.

**Table 1:** Past and present affiliations of the authors

Frank A. Barile Ph.D.: Editor of Toxicology in vitro, past president of dermal toxicology specialty section of the Society of Toxicology
Prof. Sir Colin Berry: Former chairman of the Advisory Committee on Pesticides, UK and of the Committee on Dental and Surgical Materials
Prof. Bas Blaauboer: Former editor-in-chief of toxicology in vitro
Prof. Alan Boobis: Chair of the UK Committee on Toxicity, past chair of FAO/WHO JMPR and JECFA, Past President of EUROTOX, former Editor of Food and Chemical Toxicology
Prof. Hermann M. Bolt: Former chair of the Scientific Committee of Occupational Exposure Limits, Deputy Editor-in-Chief of Archives of Toxicology, Past President of EUROTOX
Christopher Borgert Ph.D.: President & Principal Scientist Applied Pharmacology and Toxicology, Inc
Prof. Wolfgang Dekant: Former Editor-in-Chief of Toxicology Letters
Prof Daniel Dietrich: Editor of Chemico-Biological Interactions and of Computational Toxicology; Chair of Public–Private platform for the Pre-validation of testing methods on Endocrine Disruptors) Science Advisory Team; Elected external expert „Life Sciences for human well-being “ for the European Parliament
Prof Jose L. Domingo: Editor-in-Chief of Food and Chemical Toxicology
Prof. Corrado Galli: President of the Italian Society of Toxicology (SITOX)
Gio Batta Gori: Past Editor-in-Chief of Regulatory Toxicology and Pharmacology
Prof. Helmut Greim: Former chair of Scientific Committee on Health and Environmental Risks, Past President of the German Society of Pharmacology and Toxicology
Prof. Jan G. Hengstler: Editor-in-Chief of Archives of Toxicology
Prof. Pat Heslop-Harrison: Former Editor-in-Chief of Annals of Botany, Past President of Society for Experimental Biology, Vice-President European Cytogeneticists Association
Sam Kacew Ph.D.: North America Editor Toxicological and Environmental Chemistry, Associate Editor, Toxicology and Applied Pharmacology
Prof. Hans Marquardt: Former Editor of Toxicology
Prof. Angela Mally: Editor in Chief of Toxicology Letters, Member of the Committee of Contaminants and other Undesirable Substances in the Food Chain of the BfR
Prof. Olavi Pelkonen: Former Specialty Chief Editor of Frontiers in Pharmacology and of Predictive Toxicology
Prof. Kai Savolainen: Editor-in-Chief of Human and Experimental Toxicology
Prof. Emanuela Testai: Co-Editor of Toxicology Letters, Vice-chair of the Scientific Committee for Health, Environmental and Emerging Risks
Prof. Aristides Tsatsakis: Editor of Food and Chemical Toxicology, Editor-in-Chief Toxicology Reports, past President & Honorary member of EUROTOX
Prof. Nico Vermeulen: former Editor-in-Chief of Environmental Toxicology & Pharmacology, Past President of the International Society for the Study of Xenobiotics

We emphatically concur with the introductory remarks of the Guest Editorial:

*Over the past decades, there has been an unprecedented regulatory drive in public health protection in the European Union, making large-scale toxicological incidents or mass poisonings such as, e.g. the thalidomide disaster of the 1950s–60 s or the Seveso incident in 1976 an issue of the past. Although this is rarely perceived by the media and the general public, the implementation of a complex and interdependent network of regulations for chemical substances, including industrial chemicals, plant protection products, biocides, or chemicals in food and feed has minimised toxicological risks and has continuously increased public health and wellbeing in the EU. Moreover, although this framework already provides one of the most advanced regulatory systems worldwide, it is constantly pressed for improvement by scientific progress as well as an ever-increasing public awareness.*

The BfR points out that, despite several investigative efforts, there are no statistical data that support an apparent concern that in the EU chemical risk is an important or growing detrimental factor to human health.

In a carefully considered commentary, the BfR makes clear that in the necessary and continuous process of improving consumer protection any initiative taken must be based on state-of-the-art scientific knowledge and must include a broad discussion, taking into account the complex scientific, economic and societal issues involved. It is important not to confound scientific evidence for risk with other factors in determining policy.

In more detail the BfR authors address major deficiencies of the CSS proposal:

- The terms, “toxic-free” and “pollution” are not defined.
- A differentiation between hazard, exposure and risk needs to be addressed. Risk can only be quantified and managed when data on both potency and exposure provide information on whether a substance is likely to be harmful.
- No justification of the statement that “exposure to a mixture can give rise to adverse health and environmental effects, even at levels of exposure which are considered ‘safe’ for the individual chemicals on their own…” is provided. There appears to be a naïve assumption that all interactions will be additive.
- There is an implicit assumption that chemicals with an endocrine-disrupting potential are not sufficiently covered by the existing regulatory system in the EU. They do not need further regulation—all scientifically justified concerns are presently considered.

Instead of regulating non-existent or already well-considered risks, the BfR proposes that several existing faults or omissions, which slow down the regulation of dangerous chemicals should be rectified. These include,

- An ineffective and slow collection and consideration of available and relevant data.
- The time-consuming and bureaucratic procedures that delay the publication of regulations.

We also concur with further conclusions of the BfR authors:

- Thanks to the existing system of chemicals regulation in the EU, the current level of protection of its population as a whole, including sensitive sub-populations, against chemical risk is among the highest in the world. The rather bleak picture connoted in the CSS and its associated SWDs^2^ thus appears misplaced.
- A modern and enlightened society should base its decisions on the best scientific knowledge available.
- It is fully agreed that improvements to the existing regulatory procedures should be accelerated and streamlined, albeit not at the expense of scientific rigour, and that availability and accessibility of the data required for risk assessment still need to be improved significantly.

We support the BfR authors’ request that a clear science-based evaluation should be made of whether additional measures are necessary to regulate what may be irrelevant risks. Action on “public concern” in the absence of scientific justification needs to be justified on other grounds. If the public concern is evident in well-regulated areas the real risk needs to be communicated to the public more effectively. Finally, as the implementation of sustainable development is mentioned repeatedly, a clear definition and stringent requirements for implementation need to be provided by the CSS.

We hope that the critical evaluation of the CSS by the BfR will trigger a science-based debate on the different aspects addressed in the CSS. Otherwise, CSS remains a document without scientific justification.

## Footnotes

1 Present and past functions see Table 1

2 Staff Working Documents.
